# *Symbiodinium thermophilum* sp. nov., a thermotolerant symbiotic alga prevalent in corals of the world's hottest sea, the Persian/Arabian Gulf

**DOI:** 10.1038/srep08562

**Published:** 2015-02-27

**Authors:** B. C. C. Hume, C. D'Angelo, E. G. Smith, J. R. Stevens, J. Burt, J. Wiedenmann

**Affiliations:** 1 Coral Reef Laboratory. Ocean and Earth Science, University of Southampton, Waterfront Campus, National Oceanography Centre, Southampton, SO14 3ZH, UK; 2 Marine Biology Laboratory, Centre for Genomics and Systems Biology, New York University – Abu Dhabi, PO Box 129 188, Abu Dhabi, United Arab Emirates; 3 Department of Biosciences, College of Life and Environmental Sciences, University of Exeter, Exeter, EX4 4QD, UK; 4 Institute for Life Sciences, University of Southampton, Highfield Campus, Southampton, SO17 1BJ, UK

## Abstract

Coral reefs are in rapid decline on a global scale due to human activities and a changing climate. Shallow water reefs depend on the obligatory symbiosis between the habitat forming coral host and its algal symbiont from the genus *Symbiodinium* (zooxanthellae). This association is highly sensitive to thermal perturbations and temperatures as little as 1°C above the average summer maxima can cause the breakdown of this symbiosis, termed coral bleaching. Predicting the capacity of corals to survive the expected increase in seawater temperatures depends strongly on our understanding of the thermal tolerance of the symbiotic algae. Here we use molecular phylogenetic analysis of four genetic markers to describe *Symbiodinium thermophilum*, sp. nov. from the Persian/Arabian Gulf, a thermally tolerant coral symbiont. Phylogenetic inference using the non-coding region of the chloroplast *psbA* gene resolves *S. thermophilum* as a monophyletic lineage with large genetic distances from any other ITS2 C3 type found outside the Gulf. Through the characterisation of *Symbiodinium* associations of 6 species (5 genera) of Gulf corals, we demonstrate that *S. thermophilum* is the prevalent symbiont all year round in the world's hottest sea, the southern Persian/Arabian Gulf.

Shallow water coral reefs are in decline at a global scale and might be lost within this century^1^,^2^,^3^. The increasing frequency and intensity of heat stress episodes have been identified as a major threat to reefs' survival^4^,^5^,^6^. The impact of high temperature on corals can be exacerbated by regional factors such as nutrient enrichment of seawater^7^,^8^.

Corals living in symbiosis with unicellular dinoflagellate algae (zooxanthellae) belonging to the genus *Symbiodinium* are primarily responsible for the formation of tropical shallow water reefs^9^,^10^. Several environmental factors, including elevated or reduced seawater temperatures can trigger the loss of zooxanthellae from the host, a phenomenon known as “coral bleaching”^11^. Mass mortality is frequently observed among bleached corals^12^. Over the past decades, mass bleaching events, caused primarily by elevated seawater temperatures (as little as 1°C above the average annual maximum), have become more frequent and contribute to the observed degradation of coral reefs^12^,^13^.

Coral-*Symbiodinium* associations exhibit some capacity for acclimatization/adaptation to elevated temperatures^14^,^15^,^16^,^17^. In a number of locations in the Persian/Arabian Gulf (hereafter referred to as ‘the Gulf’) corals are able to cope with exceptionally high seasonal temperature maxima (34–36°C) as well as large (~20°C) annual fluctuations^18^,^19^,^20^,^21^. The existence of these coral communities indicates that at least some coral-*Symbiodinium* associations can survive under conditions that are predicted to occur in coral reef containing waters elsewhere in the next 100 years. The heat tolerant Gulf coral populations have established themselves within a relatively short period of less than ~15,000 years, after the gulf basin was flooded^22^,^23^. Hence, they represent ideal models to study the basis of heat stress tolerance and the adaptive capacity of reef corals, contributing essential information required to forecast the fate of coral reefs in the warmer oceans of the future^21^. However, the physiological basis for this stress resilience is not yet understood^24^.

One strategy of corals to cope with high sea surface temperatures (SSTs) is to host populations of thermally tolerant *Symbiodinium* *spp.*^11^,^25^,^26^,^27^. The prevalence of *Symbiodinium* clade D, considered to convey an increased heat stress tolerance, was reported in corals collected in the northwestern Gulf off the Saudi Arabian coast and off the coast of Iran^25^,^28^,^29^ (Fig. 1). It was inferred from these results that the association with clade D *Symbiodinium spp*. may partially explain the thermal resilience of Gulf corals^25^. Apart from permanent associations with clade D symbionts, transient ‘symbiont shuffling’ can potentially increase the heat tolerance of corals^25^,^26^,^30^.

Unexpectedly, sequencing of the ITS2 region of the nuclear ribosomal DNA (nrDNA) did not detect *Symbiodinium* clade D in six common coral species from the extreme temperature habitat of the southern Gulf^21^. Instead, nearly 60% of the analysed sequences identified ITS2 type (subclade) C3 as the prevalent zooxanthellae in this region. This result was surprising since subclade C3 is considered a cosmopolitan, thermally sensitive and host generalist symbiont^31^.

To characterise the significance of Gulf ITS2 type C3 *Symbiodinium* (hereafter ‘Gulf C3’) in the functioning of coral-*Symbiodinium* associations in the extreme temperature environment of the Gulf, two important questions need to be answered: Firstly, is the prevalence of Gulf C3 in the southern Gulf a temporal phenomenon that might be reverted seasonally or permanently to a dominance of clade D symbionts? Secondly, does Gulf C3 represent a lineage unresolved by ITS2 type phylotyping and genetically distinct from ITS2 type C3 *Symbiodinium* found exterior to the Gulf?

To assess the temporal variability of Gulf coral-*Symbiodinium* associations we monitored the seasonal dominance of symbiont types (resolved using the ITS2 region) in tagged colonies of six coral species over 22 months. During this analysis an ITS2 C3 variant was detected in the Gulf C3 samples from *Porites* spp. and *Platygyra daedalea*. To assess whether this variant was indicative of a genetically distinct lineage, the phylogenetic resolution of Gulf C3 was further assessed through analysis of the highly variable non-coding region of the chloroplast *psbA* gene (*psbA^ncr^*). This marker was recently used to demonstrate that many lineages within ITS2 type C3 represent distinct *Symbiodinium* species^32^. Furthermore, we included the domain V of the chloroplast large subunit ribosomal DNA (cp23S) and the mitochondrial cytochrome b gene (*cob*) in the phylotype characterisation of Gulf C3-containing coral populations along >400 km of coastline in the Gulf. We propose that Gulf C3 as resolved by the ITS2, cp23S, *cob and psbA^ncr^* markers represent a new species that we have named *Symbiodinium thermophilum*. Despite some species-specific variability in seasonal composition of the symbiont complement, *Symbiodinium thermophilum* represents the predominant symbiont of corals from the southern Gulf, the world's hottest sea.

## Results

### Seasonal variation of coral-*Symbiodinium* associations

To assess the potential seasonal variability in the complement of dominant zooxanthellae types, we studied six common species of scleractinian corals from Saadiyat reef in the Southern Gulf. Per species, three tagged colonies were repeatedly sampled over the period from June 2011 to March 2013; a total of 138 data points (Fig. 2; Supplementary Table S1 & Fig. S1). Denaturing gradient gel electrophoresis (DGGE) of the nrDNA ITS2 region was used to identify predominant *Symbiodinium* types associated with the corals. Cloning of predominant bands within DGGE fingerprints was used to unambiguously identify the represented phylotypes (Fig. 2). We detected the presence of A1, C3, a novel C3 variant (1 bp different from ITS2 type C3, referred to here as C3v1; deposited in Genbank accession number KM487747), C1, C15, D1-4 (characterised by the presence of D1 and D4 ITS2 sequences *sensu* Lajeunesse et al. 2010^27^; now formally described as *S. trenchii*^33^) and a novel G3 variant (one bp different from ITS2 type G3, deposited in Genbank accession number KM487749).

A predominance of C3-*Symbiodinium* in the southern Gulf is demonstrated by its detection in 89% of all data points (Supplementary Fig. S1). Most notably, *Porites harrisoni* and *Porites lutea* contained exclusively C3 type *Symbiodinium* as their dominant zooxanthellae throughout the 22 month period (Fig. 2). In contrast, *F. pallida* showed a more varied zooxanthellae complement (mostly C3, C15 and A1) that was relatively stable over time (Fig. 2, Supplementary Fig. S1). *Acropora downingi* colonies predominantly (present in all but one *A. downingi* data point) harboured the novel ITS2 type C3v1. This C3 variant was not found in the other corals.

With the exception of *Porites* spp., the studied species showed signs of seasonal variability with the zooxanthellae complement becoming more varied during the cold months. However, clade D was detected only in one winter data point in *C. microphthalma* (Fig. 2; Supplementary Fig. S1).

### Phylogenetic resolution of Gulf C3 using the cp23S, *cob* and *psbA^ncr^* markers

**Figure 1:**
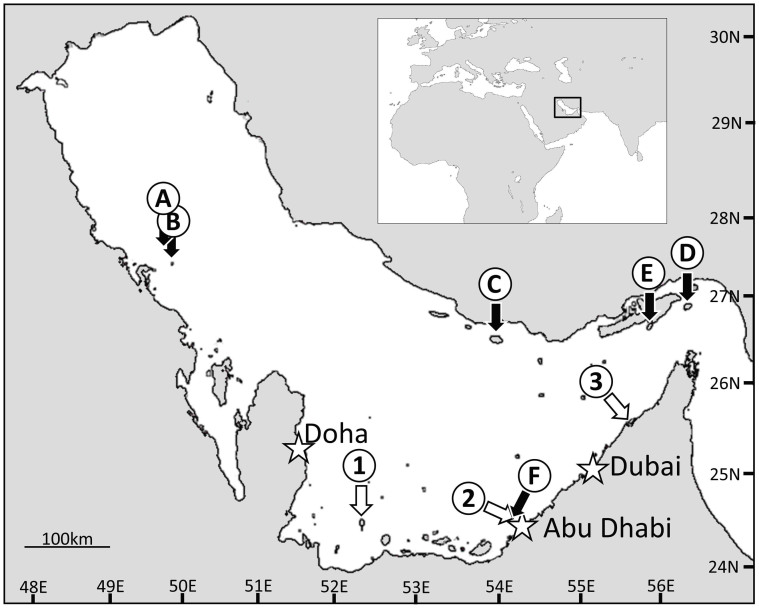
Coral-*Symbiodinium* sampling locations within the Persian/Arabian Gulf. (A-E) represent previous sampling locations from Baker et al. 2004^25^ (A&B), Mostafavi et al. 2007^28^ (C&D), Shahhosseiny et al. 2011^29^ (E) and Hume et al. 2013^21^ (F). 1–3 represent sampling locations from this study: Dalma (1), Saadiyat (2) and Umm Al Quwain (3). Major cities are marked with a ⋆. Both the main and insert map were created from a mosaic of LandSat 8 OLI/TIRS “Natural Colour” images acquired through the USGS's EarthExplorer (http://earthexplorer.usgs.gov/). Images were collated in GNU Image Manipulation Program (GIMP) 2.6.11 (www.gimp.org). Land-Sea boundaries were identified and land and sea masses were coloured to contrast, also in GIMP. Latitude and Longitude markers were added manually along with sampling locations, map scale, and major city locations.

Our temporal analysis of the zooxanthellae complement suggests *Porites* spp. as an ideal model to evaluate the taxonomic status of Gulf C3 since the zooxanthellae complement is dominated by this symbiont year-round. Therefore, we collected 22 coral colonies, of three species (*P. lobata, P lutea and P. harrisoni),* from three sites (Dalma, Saadiyat and Umm Al Quwain; Fig. 1) that spanned >400 km of coastline in the Southern Gulf. We used DGGE analysis of the ITS2 region in combination with cloning and sequencing and confirmed that the zooxanthellae complements of these corals were also dominated by ITS2 type C3 *Symbiodinium.* During this analysis a novel C3 variant sequence that contained an 8 bp duplication (Supplementary Fig. S2; accession number KP234524) was detected among the standard C3 sequences. The presence of this duplication was further investigated in an additional 18 corals (Supplementary Table S2). The 25 coral colonies analysed returned 238 ITS2 sequences. Of these, 63 sequences (~27%) from 15 different coral specimens contained the ITS2 C3 variant sequence (termed C3-Gulf ITS2 variant). The presence of a single band containing both C3 and C3-Gulf ITS2 variant sequences in the DGGE analysis demonstrated that the two sequences were irresolvable in this DGGE analysis. An analysis of >830 ITS2 type C3 sequences available in databases found this specific 8-bp duplication in only one entry from *Symbiodinium* of a Red Sea *Stylophora pistillata* (Genbank JX048681.1).

To assess for the presence of lineages potentially unresolved by the ITS2 nrDNA region (as signified by the Gulf-specific C3-Gulf ITS2 variant), we amplified and sequenced regions of the cp23S, *cob* and *psbA^ncr^*. These sequences were then subjected to molecular phylogenetic analyses in comparison with sequences from ITS2 type C3 *Symbiodinium,* or closely related variants, from both the Indo-Pacific and Atlantic basins available from databases (Figs. 3 & 4; see Supplementary Tables S3–S5 for a list of accession numbers relating to *psbA^ncr^,* cp23S and *cob* sequences generated and used in this study).

Of the 12 corals phylotyped with the cp23S and *cob* markers a single haplotype was obtained for each marker (accession numbers KP234523 & KP234522). Within the cp23S phylogeny (Fig. 4) Gulf C3 grouped with subclades C1, C78a, C90, C3nt, C79, C42a and C8a. Such a grouping of 7 out of the 14 subclades analysed demonstrates the resolving power of the cp23S marker to be between the cladal and subcladal (ITS2) level. Resolving closer to the subcladal level, the *cob* marker resolved Gulf C3 as genetically distinct from all other analysed subclades (Fig. 4). Both phylogenies resolved the Gulf C3 as genetically distinct from the ITS2 C3 sample included in the analysis (collected from Heron Island on the Great Barrier Reef; accession numbers FJ529546 & FJ529535).

Phylogenies inferred from *psbA^ncr^* sequences resolved Gulf C3 *Symbiodinium* as a well-supported (PP = 1.00) distinct monophyletic lineage with a large genetic distance to all other C3, or closely related ITS2 type *Symbiodinium* variants (Fig. 3a&b; Supplementary Fig. S3). The genetic distance between the *psbA^ncr^* sequences of the Gulf C3 sample BH1451 (see Supplementary Fig. S3) and the closest related non-Gulf C3 type (accession KF572379; genetic distance = 0.236) was substantially larger than that observed between most distant representatives of the most distantly related (according to ITS2 sequences) ITS2 types included in the analysis (C27-JQ043674 and C40-KF572369; 5 bp difference in ITS2 sequence; pairwise genetic distance = 0.151) (Fig. 3c).

The groupings of the *psbA^ncr^* sequences from ancestral C3 and more derived ITS2 type *Symbiodinium* accorded with previous phylogenetic analyses of the *psbA^ncr^*^32^,^34^.The majority of more derived ITS2 types resolved as monophyletic groupings (e.g. C3a, C3h, C3k, C7, C7c, C27, C87, C31/C31c & C40), with the exceptions of C7a and C21 which resolved as paraphyletic, and C3b as polyphyletic (Fig. 3a).

Within-group and between-group pairwise genetic distances of *psbA^ncr^* sequences were calculated to further characterise the Gulf C3 lineage in relation to other ITS2 types. The mean within-group genetic difference (for those ITS2 types with >10 *psbA^ncr^* sequences available) of the Gulf C3 lineage was lower than the majority of ITS2 types with only types C31 and C40 demonstrating lower distances (0.015 & 0.020, respectively; Gulf C3 average genetic difference = 0.022; Fig. 3c). Between-group genetic distances between the *psbA^ncr^* sequences of two of the most distantly related ITS2 types (C27 and C40) were considerably lower than between the Gulf C3 type and their closest lineage, C3b (0.140 and 0.248, respectively).

### Formal description of *Symbiodinium thermophilum* sp. nov

Based on inferred molecular phylogenies using the ITS2, cp23S, *cob* and *psbA^ncr^* and prevalence in a habitat with extreme environmental conditions, we describe a new species within the genus *Symbiodinium*.

#### Diagnosis

Nucleotide sequences of the nrDNA ITS2 region type C3 and C3-Gulf ITS2 variant (Genbank accession numbers KM487748 & KP234524), cp23S (Genbank accession number KP234523), *cob* (Genbank accession number K234522) and characteristic chloroplast *psbA^ncr^* sequences (Genbank accession number KM458273-KM458294) found in zooxanthellae with a temperature stress resilient phenotype and prevalence in extreme temperature/high salinity environments define this species.

#### Holotype designation

Preserved collection of zooxanthellae extracted from a *P. lobata* colony and submitted to the Natural History Museum London, UK (Registration number BM000794154).

#### Type locality

Collected from the scleractinian coral *P. lobata* from 4 m depth on Saadiyat reef in Abu Dhabi, United Arab Emirates (2435056.400N, 5425017.400E).

#### Etymology

**“***thermophilum”* referring to the thermally resilient phenotype of the species.

## Discussion

The reporting of prevalent C3 associations in 7 species of Gulf corals by Hume et al. 2013^21^, contrasted the findings of a predominance of Clade D *Symbiodinium* elsewhere in the Gulf^25^,^28^,^29^ (Fig. 1). However, it was unclear as to whether this finding was a true representation of the predominant coral-*Symbiodinium* associations of the Southern Gulf or a temporal phenomenon. Here we demonstrate that despite a species-specific tendency to host a more varied zooxanthellae complement during the cold months of the year, Gulf C3 (and derived variants) remain the predominant symbiont in corals from the southern Gulf.

**Figure 2:**
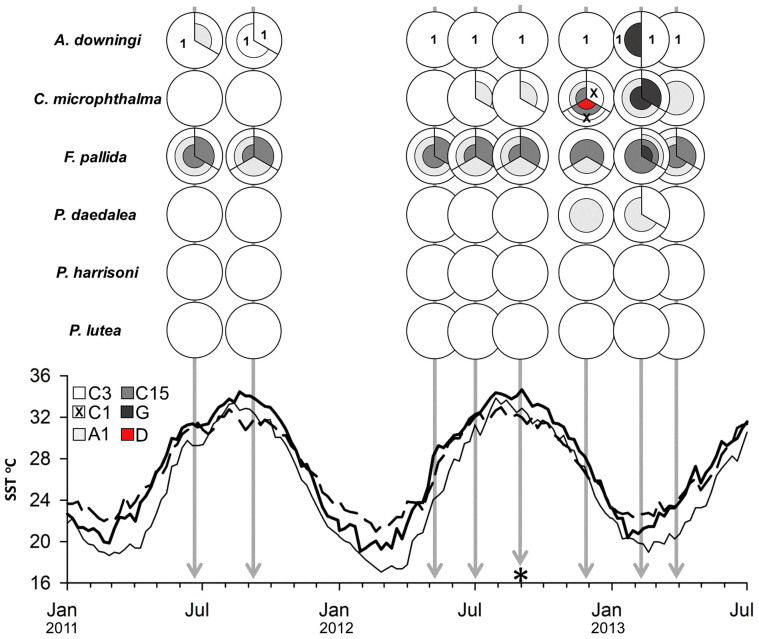
Relation of predominant *Symbiodinium* type to seasonal thermal profiles. Top) Classification of predominant *Symbiodinium* type associated with 6 species of southern Gulf (Saadiyat) corals at each of 8 seasonal time points. Sampling date is denoted by position along the x axis of the plot below. * denotes the time point at which coral bleaching was visually observed on the reef during sample collection. Each pie chart represents the number of times a given symbiont (for example C3), or mix of symbionts (for example a mix of C3 and A1) was detected in a coral colony (symbiont mixes identified by the vertical division of a slice; the proportion of each symbiont making up a mix was not quantified). Novel ITS2 variant C3v1 is denoted by a 1 within a C3 section of chart. Further details of coral phylotyping can be found in Supplementary Table S1. Bottom) Plot of remotely sensed sea surface temperatures (SSTs) for waters in close proximity to the sampling sites of Baker et al. 2004^25^ (Jana island; 27.3N 49.9E;–), Mostafavi et al. 2006^28^ (Larak island; 26.8N 56.4E;---) and this study (Saadiyat; 24.7N 54.4E;–). SST data were obtained from the GHRSST Level 4 K10_SST Global 1 meter Sea Surface Temperature Analysis dataset (http://www.nodc.noaa.gov/SatelliteData/ghrsst/accessdata.html).

The detection of clade D at only one winter data point during this study is in line with the absence of this clade among the samples collected in summer by Hume et al.^21^. These data demonstrate that clade D symbionts are not responsible for the exceptional thermal resilience of corals in the southern Gulf. Clade D symbionts, specifically *S. trenchii*, have been characterised as opportunistic symbionts^33^,^35^,^36^, replacing previously dominant zooxanthellae strains lost from coral colonies during bleaching. These changes (e.g. C3 to D1-4^26^) might result in increases in thermal resilience, however, tradeoffs in energetic and reproductive fitness for the host have also been demonstrated^30^. Despite potential colonisation opportunities that might have been created by the bleaching of reefs off the coast of Abu Dhabi in 2010^37^ and 2012 (personal observation of the authors) type C3 remains predominant. The reason for this lack of clade D in the southern Gulf is unclear, however, reefs of the southern Gulf do experience higher summer temperatures and salinity levels^38^ (Fig. 2) than the reefs previously sampled in the northwest^25^ and northeast^28^,^29^ of the Gulf where clade D is predominant. In addition to these high temperatures, low temperature extremes of the Gulf have the potential to significantly stress coral-*Symbiodinium* associations in winter months^39^,^40^,^41^. Therefore, the rarity of clade D in the southern Gulf might suggest that the unique physicochemical conditions in these waters may limit the proliferation of clade D and promote the prevalence of associations with C3-type *Symbiodinium*.

**Figure 3:**
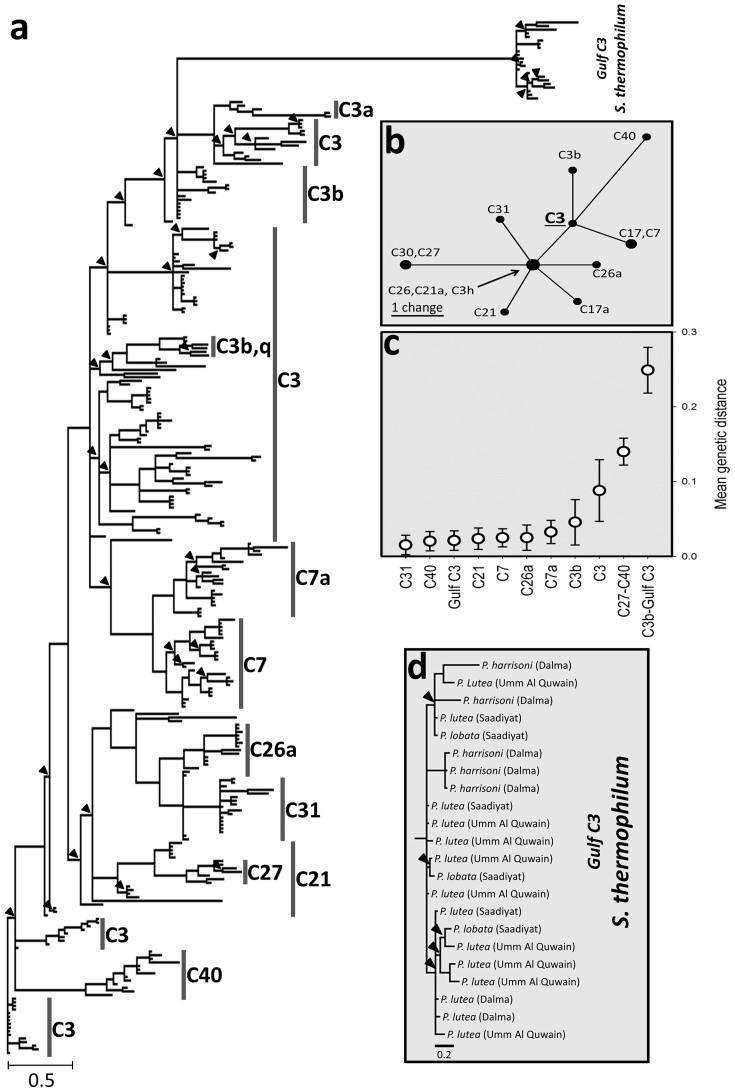
Resolution of phylogenies within ITS2 type C3 and closely related variants using the ITS2 and *psbA^ncr^* markers. (a) High resolution phylogeny of Gulf and non-Gulf ITS2 type C3 and closely related ITS2 type variants as estimated through Bayesian Inference of the chloroplast *psbA^ncr^*. Sequences are annotated according to their ITS2 type. Gulf ITS2 type C3 sequences are annotated *S. thermophilum* as described in this report. Support for nodes is assessed using posterior probabilities (PP) presented on the tree as follows: 0.75-1.00, no annotation; 0.5-0.75, marked by (▴). No nodes had a support of less than 0.5. The tree is rooted according to an ITS2 type C3 sequence collected at the Great Barrier Reef (accession JQ043643). The tree showing the full details of node support and sample accession numbers is shown in Supplementary Fig. S2. Full details of the sequences used in the phylogenetic estimation are available in Supplementary Tables S3 and S4. (b) Maximum parsimony ITS2 haplotype network of the ITS2 types included in the phylogenetic analysis. (c) Within and between-group mean pairwise genetic differences with error bars (standard deviation). (d) Magnified region of the *psbA^ncr^* tree including *S. thermophilum* samples annotated with host and location sampling information.

**Figure 4:**
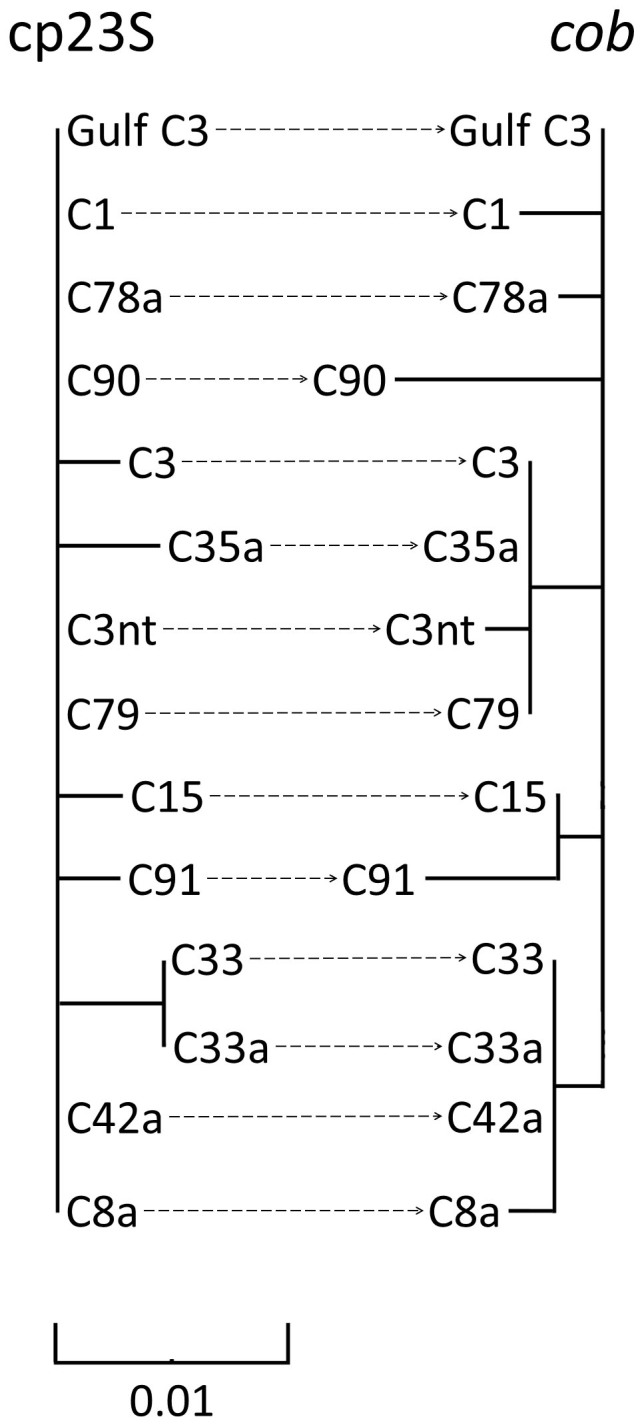
Unrooted Bayesian inferred phylogenies of ITS2 type C3 and closely related variants using domain V of the chloroplast large subunit ribosomal DNA (cp23S) and the mitochondrial cytochrome b gene (*cob*). Support of nodes was assessed using posterior probabilities (PP). PPs above 0.75 are not displayed. Both trees are drawn to the same scale and analysed sequences are annotated according to their ITS2 type. See Supplementary Table S5 for a list of sequences used in both inferences.

The presence of a novel ITS2 C3 variant sequence, C3-Gulf ITS2 variant, identified in this study could be interpreted as either an intragenomic variant of the Gulf C3 symbiont or alternatively could represent the presence of two ITS2 type C3 symbionts within the Gulf corals containing it. However, the relatively low *psbA^ncr^* within group pairwise genetic distances of Gulf C3 (lower than that of the other ITS2 types; see Fig. 3c) and the single phylotype sequences returned from direct sequencing of *psbA^ncr^* PCR products would suggest the former to be true. Despite the low abundance of this ITS2 sequence variant, its regular occurrence only among sequences from zooxanthellae of Gulf-derived corals qualifies it as a taxonomic marker for regional Gulf C3 populations^42^. However, given that this sequence variant was not detected in 10 of the 25 analysed corals (Supplementary Table S2) the ITS2 region alone cannot be relied upon to resolve Gulf C3.

The prevalence of ITS2 type C3 *Symbiodinium* in the extreme conditions of the Gulf contradicts the current phenotypic classification of C3 type zooxanthellae and closely related variants as thermally sensitive symbionts^31^ and could imply that this type has a much greater phenotypic plasticity than previously thought. However, the occasional presence of a specific ITS2 sequence variant suggests that the ITS2-based molecular taxonomy may not resolve the genetic diversification that has led to different physiological capacities. Such a lack of phylogenetic resolution could be due to concerted evolution acting on the ITS2 C3 type^32^,^34^,^43^,^44^,^45^. Since the nrDNA gene exists as multiple copies (highly variable copy numbers within and between clades ranging from 100 s to 10000 s^46^) in the *Symbiodinium* genome, concerted evolution could have restricted rare ITS2 sequence variants (such as C3-Gulf ITS2 variant found in this study) from becoming numerically dominant in species lineages that diverged millions of years ago^32^. The genetic differentiation of Gulf C3 and non-Gulf ITS2 type C3 resolved using the cp23S and *cob* markers (both of which resolve above the subcladal/ITS2 level) further demonstrates the potential evolutionary masking by the multi-copy ITS2 region through concerted evolution.

The genetic distance between the two most closely related *psbA^ncr^* haplotypes from Gulf and non-Gulf ITS2 type C3 *Symbiodinium* is larger than that between the *psbA^ncr^* sequences from sequences representing the two most distantly related ITS2 type variants (C27/C40) (Fig. 3c). Our results therefore support the hypothesis that concerted evolution might impede sequence divergence within ITS2 type C3 *Symbiodinium* and ‘mask’ pronounced genetic diversification^32^,^34^,^44^,^45^. Indeed, the fact that the *psbA^ncr^* marker sequence resolves Gulf C3 with such large between-group genetic distances indicates this to be an exceptionally strong case of masking by the ITS2 genetic marker.

Thornhill et al. 2014^32^ analysed a combination of genetic markers including the ITS2 region, *psbA^ncr^* sequences and microsatellite loci to demonstrate sexual recombination boundaries between, and sexual recombination within, the sympatric ITS2/*psbA^ncr^* lineages C7 and C7a, thus demonstrating species level resolution of the *psbA^ncr^* marker according to the biological species concept^47^. The within-group genetic distances among *psbA^ncr^* sequences of Gulf C3 are smaller than those of the C7 and C7a type lineages/species. This result, in combination with the substantially large between-group distance of Gulf C3 and other C3 and C3 variant types would suggest that the Gulf C3 *Symbiodinium* is a sexually isolated lineage within the ITS2 C3 and closely related types and therefore, would likely satisfy the biological species concept^47^,^48^. The lack of shared ancestral *psbA^ncr^* haplotypes between Gulf C3 and any non-Gulf C3 sample as well as the genetic differentiation between Gulf C3 and non-Gulf C3 *Symbiodinium* by the cp23S and *cob* marker qualifies Gulf C3 as a species according to the phylogenetic species concepts^47^,^48^. The definition of this species by molecular phylogeny is supported by its prevalence in a unique ecological niche or adaptive zone (an extreme temperature habitat with high salinity waters) and thus also satisfies the ecological species concept^47^,^48^,^49^. We therefore formally describe here *S. thermophilum*.

The Gulf represents a young sea that was formed ~15 k years ago and shifted towards the present temperature regime only since ~3–6 k years^22^,^23^. If the origin of *S. thermophilum* is in the Gulf, one would need to assume a very rapid evolution of its distinct genetic and physiological traits. Alternatively, *S. thermophilum* may have originated from a numerically rare, pre-adapted population originally present in waters outside of the Gulf which has since proliferated in the extreme environment of the Southern Gulf.

The acclimation potential of corals may not be sufficient to keep up with the increasing ocean temperatures and models project that coral reefs globally will experience annual bleaching by 2040^30^,^50^. In this context, the symbionts of the Gulf represent a genetic resource that could potentially facilitate the necessary increases in thermal tolerance for corals to withstand temperatures predicted towards the turn of the century^21^. However, it is unknown whether genetic material may be naturally exported from the Gulf to adjacent waters against the prevalent surface inflow current^19^ or whether these specialised associations will be able to survive in the less saline and cooler waters of the wider Indo-Pacific. These knowledge gaps as well as the elucidation of the mechanisms underlying the thermal resilience of Gulf associations must be addressed urgently.

## Methods

### Sample collection and nucleic acid extraction

In order to assess for seasonal variation in Gulf coral-*Symbiodinium* associations, 6 species of coral were monitored for a period of 22 months. Three fragments of coral (approximately 4 cm^2^) were removed with hammer and chisel from three colonies each of *Acropora downingi*, *Cyphastrea microphthalma*, *Favia pallida*, *Platygyra daedalea*, *Porites harrisoni* and *Porites lutea* at Saadiyat reef, off the coast of Abu Dhabi on the 21.06.11. The replicate samples of each colony were only processed for time points or species when exceptional variation was determined after initial analysis of the first replicates (Supplementary Table S1). After collection, fragments were placed directly into absolute ethanol. Fragments were removed from the sides of the colonies, all of which were in 5–7 m depth of water. Colonies were tagged and later sampled on the following dates: 07.09.11, 08.05.12, 01.07.12, 27.08.12, 25.11.12, 10.02.13 and 28.03.13. To further assess unresolved lineages within Gulf C3 using the *psbA^ncr^*, cp23S and *cob* markers, samples of *P. lobata, P. lutea and P. harrisoni* were collected from Saadiyat reef, Dalma Island and off the coast of Umm Al Quwain in September 2012 (Dalma and Saadiyat) and March 2013 (Umm Al Quwain) (Supplementary Table S4). After removal from the coral, tissue samples were immediately fixed in absolute ethanol or frozen on dry ice. Additional cloning of the zooxanthellae ITS2 region of the nrDNA was performed on a subset of the corals collected in September 2012 and March 2013 and also on a selection of corals collected in October 2011 (as described in Hume et al., 2013^21^) and from Gulf-originating (collected from Saadiyat reef, May 2010), aquaria cultured, *P. lobata* colonies now kept at the Coral Reef Laboratory Experimental Mesocosm, Southampton, UK^51^ (sampled June 2014; Supplementary Table S2). Genomic DNA of all samples was extracted using a previously described CTAB-based protocol^21^. DNA was dissolved in deionized water and stored frozen at −20°C.

### DGGE analyses

To determine dominant *Symbiodinium* types within the six species of Gulf corals, the ITS2 region was analysed by DGGE-PCR as detailed in Supplementary Information. In short, the ITS2 region was amplified from genomic DNA template by PCR using the primer pair SYM_VAR_5.8SII and SYM_VAR_Clamp. DGGE analysis was conducted using a BioRad DCode System for DGGE with a model 475 gradient former. Samples were run on a 32.5–57.5% gradient for 1400 Vh. The banding patterns produced on the gels for each sample, known as a ‘fingerprint’ are characteristic of a given *Symbiodinium* type or mix of types. The use of pre-characterized markers allows the identification of subcladal type according to the distance bands run in the DGGE gel (Supplementary Fig. S4). By extracting and sequencing novel bands a library of known fingerprint-type associations is built and can be used to assess future samples.

### Amplification and sequencing of the ITS2, *psbA^ncr^*, cp23S and *cob* regions

Twenty-two samples that had their dominant symbiont phylotyped (directly sequenced PCR product) using the *psbA^ncr^* marker. Twelve of these samples additionally had regions of the zooxanthellae cp23S and *cob* markers amplified, cloned and sequenced (Supplementary Table S4 and see Supplementary Table S6 for PCR reaction conditions and primer details). Additional cloning and sequencing of the nrDNA ITS2 region was conducted on 7 of the 22 *psbA^ncr^* analysed samples as well as on an additional 18 coral samples (summarised in Supplementary Table S2).

ITS2 PCR products were cloned using the Strataclone PCR Cloning Kit (Agilent Technologies). PCR inserts were verified by colony PCR and PCR-containing plasmid preps were made using the GeneJET Plasmid Miniprep Kit (Thermo Scientific). Sequencing services were provided by Eurofins MWG Operon with internal primer psbA_int_Fwd, 5′ CTAGGTATGGA AGTGATGCATG3′, used to sequence *psbA^ncr^* PCR products directly and universal primers T3 (ITS2, cp23S, *cob*) and T7 (cp23S, *cob*) used to sequence the ITS2-, cp23S- and *cob*-containing plasmids.

*S*equence chromatograms were checked by eye for potential sequencing errors. Any sequence with a chromatogram characteristic of multiple PCR products (for example where several peaks were registered for a single nucleotide position or where reading frame shifts were apparent) was discarded from further analysis.

### Analysis of nrDNA ITS2 region sequences

nrDNA ITS2 region sequences obtained during this study underwent screening during analysis. Chromatograms of ITS2 sequences were checked by eye for potential mis-calls or sequencing errors. Sequences were then aligned using ClustalW in Mega6^52^. In order to remove potential PCR artefacts the ITS2 sequences were screened in the following way. Any nucleotides that differed from the consensus sequence (either indels or substitutions) were reverted to the consensus nucleotide at that given position in the alignment unless the same divergence (same specific nucleotide substitution or indel) was found in a sequence resulting from a different PCR from either the other ITS2 PCR products molecularly cloned in this study or others, or ITS2 accessions in GenBank. All ITS2 sequences were analysed using the online BLAST tool provided by the National Centre for Biotechnology Information (http://blast.ncbi.nlm.nih.gov/). Any sequences that did not have 100% matches over the entire length of the ITS2 region after undergoing the above screening were considered novel ITS2 sequences.

### Creation of the maximum parsimony ITS2 haplotype network

*psbA^ncr^* sequences representing 19 different ITS2 types were included in the phylogenetic analysis (Supplementary Table S3). Of these 19 associated types, 13 types had representative ITS2 sequences available from Genbank and could be aligned using ClustalW in MEGA 6^52^. The resulting ITS2 alignment was checked by eye and haplotype networks were created using Network 4.612 (fluxus-engineering.com) using a reduced median network calculation and optional post-processing MP calculations. Several of the ITS2 haplotypes (which exhibited different *psbA^ncr^* haplotypes) grouped together in the network due to no difference in their ITS2 sequences. These are likely variants identified by DGGE that are either defined by multiple ITS2 copy variants or according to a difference in the 5.8S region rather than the aligned ITS2 region.

### Estimation of phylogeny using Bayesian Inference

In order to better resolve potentially hidden lineages within the Gulf C3 type, 22 *psbA^ncr^* sequences obtained from Gulf *Porites* samples were analysed (Supplementary Table S4) along with 264 reference sequences (Supplementary Table S3), all of which had associated ITS2 types, generated from two previous studies^32^,^34^. The *psbA^ncr^* alignment is available from the Dryad Digital Repository (http://doi.org/10.5061/dryad.q75p2). Two of the sequences from this data set (C3 representative KF572363 and C30 representative JQ043605) caused two otherwise strongly resolved monophyletic groupings (C40 and C31/C31c, respectively) to resolve paraphyletically and were considered to have erroneous ITS2 types associated with them and were therefore removed from the study. A subset of the 22 *psbA^ncr^* phylotyped corals had zooxanthellae cp23S and *cob* markers amplified (Supplementary Table S4). Each group of marker sequences was aligned using Clustal Omega^53^, prior to checking and final adjustment by eye. Only partial sequences of the *psbA^ncr^* were available for reference sequences; Gulf *psbA^ncr^* sequences were cropped accordingly. The *psbA^ncr^* alignment was submitted to the Dryad database (datadryad.org). Phylogenies were estimated by Bayesian Inference using MrBayes 3.2.2^54^ applying the Jukes-Cantor model with a gamma shaped distribution with invariable sites (*psbA^ncr^*), the Hasegawa-Kishino-Yano model (cp23S) and the Hasegawa-Kishino-Yano model with a gamma distribution (*cob*) (selected according to Akaike Information Criterion using MEGA6). The MCMC analyses were run for 2.0 × 10^6^ generations, sampling every 1000 generations for the *psbA^ncr^* analysis and 1.0 × 10^6^ generations, sampling every 500 generations for the cp23S and *cob* inferences. Convergence of chains occurred within the first 1.0 × 10^5^ generations. A relative burn-in of 0.25 was used in calculating a 50% majority rule consensus tree. A non-Gulf C3 type *psbA^ncr^* sequence collected in the Great Barrier Reef was used as an outgroup (accession number JQ043643) for the *psbA^ncr^* inference. cp23S and *cob* phylogenies were unrooted. Support of nodes was assessed using posterior probabilities (PP).

### Calculation of genetic distances

Pairwise genetic distances between *psbA^ncr^* haplotypes were calculated with MEGA 6 using all substitutions and with gaps considered via pairwise deletion. The Jukes-Cantor model with a gamma shaped distribution was used. Mean within- and between-group (grouped according to ITS2 type) genetic distances were then calculated from the pairwise differences.

## Author Contributions

J.W. conceived the study and designed experiments, analysed the data, contributed materials and wrote the paper. B.C.C.H. designed and performed experiments, contributed materials, analysed the data and wrote the paper. C.D.'A. conceived the study, analysed data and wrote the paper. E.G.S. performed experiments and contributed materials. J.R.S. designed experiments and contributed analysis tools. J.B. conceived the study, designed and performed experiments and contributed materials. All authors discussed the results and reviewed the manuscript.

## Supplementary Material

Supplementary Information Supplementary Information
