# Supplementary material for: Symbiodinium thermophilum sp. nov., a thermotolerant symbiotic alga prevalent in corals of the world's hottest sea, the Persian/Arabian Gulf
Source: Sci Rep. 2015 Feb 27;5:8562. doi: 10.1038/srep08562 (PMC4342558; doi:10.1038/srep08562)
Supplement: Supplementary Information [file srep08562-s1.pdf]

Symbiodinium thermophilum sp. nov., a thermotolerant symbiotic alga prevalent in corals of the world's hottest sea, the Persian/Arabian Gulf

Hume, B.C.C.[1]; D'Angelo, C. [1]; Smith, E.G. [2]; Stevens, J.R. [3], Burt[2], J. & Wiedenmann, J. [1,4*]

[1] Coral Reef Laboratory. Ocean and Earth Science, University of Southampton, Waterfront Campus, National Oceanography Centre, Southampton, SO14 3ZH, UK

[2] Marine Biology Laboratory, Centre for Genomics and Systems Biology, New York University – Abu Dhabi, PO Box 129 188, Abu Dhabi, United Arab Emirates

[3] Department of Biosciences, College of Life and Environmental Sciences, University of Exeter, Exeter, EX4 4QD, UK

[4] Institute for Life Sciences, University of Southampton, Highfield Campus, Southampton, SO17 1BJ, UK

Supplementary Information

Supplementary Figures

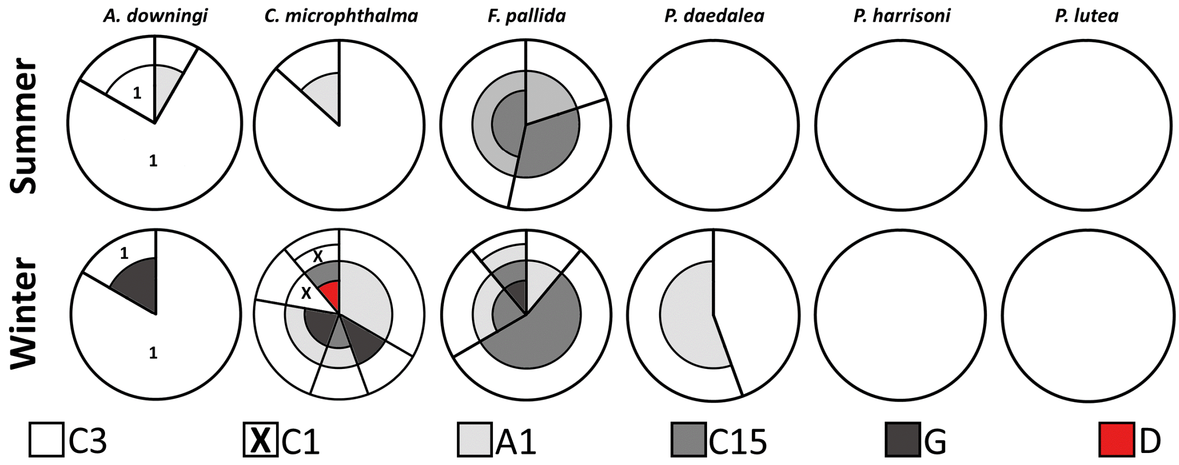

Supplementary Figure S1. Classification of predominant *Symbiodinium* types associated with 6 species of southern Gulf corals using denaturing gradient gel electrophoresis of the ITS2 region of the nrDNA. The novel ITS2 variant of the C3 type, C3v1 is denoted by a 1 within a C3 section of pie. Three coral colonies were sampled per species at each of 8 time points from 06/11-03/13. Each pie chart represents the number of times a given symbiont (for example C3), or mix of symbionts (for example a mix of C3 and A1) was detected (symbiont mixes identified by the vertical division of a slice; the proportion of each symbiont making up a mix was not quantified) in either summer (5 time points; 06/11, 09/11, 05/12, 07/12 and 08/12) or winter (3 time points; 11/12, 02/13 and 03/13) time point collections. A full breakdown of DGGE results for individual corals and replicates is available in Supplementary Table S1.

```
C3        TTGCCCCAACTTTGCAAGCAGGATGTGTTTCTGCCTTGCGTTCTTATGAGCTATTGCCCT  60
          ||||||||||||||||||||||||||||||||||||||||||||||||||||||||||||
C3 Gulf   TTGCCCCAACTTTGCAAGCAGGATGTGTTTCTGCCTTGCGTTCTTATGAGCTATTGCCCT  60

C3        CTGAGCCAATGGCTTGTTAATTGCTTGGTTCTTGCAAAATGCTTTGCGCGCTGTTATTCA  120
          ||||||||||||||||||||||||||||||||||||||||||||||||||||||||||||
C3 Gulf   CTGAGCCAATGGCTTGTTAATTGCTTGGTTCTTGCAAAATGCTTTGCGCGCTGTTATTCA  120

C3        AGTTTCTACCTTCGTGGTTTTACTTGAGT--------GACGCTGCTCATGCTTGCAACCG  172
          |||||||||||||||||||||||||||||        |||||||||||||||||||||||
C3 Gulf   AGTTTCTACCTTCGTGGTTTTACTTGAGTGACGCTGCGACGCTGCTCATGCTTGCAACCG  180

C3        CTGGGATGCAGGTGCATGCCTC  194
          ||||||||||||||||||||||
C3 Gulf   CTGGGATGCAGGTGCATGCCTC  202
```

Supplementary Figure S2. Alignment of representative ITS2 type C3 and C3-*Gulf* nrDNA ITS2 sequences. The 8bp duplication region specific for C3-*Gulf* is highlighted in magenta. The corresponding region in the C3 reference sequence is marked black.

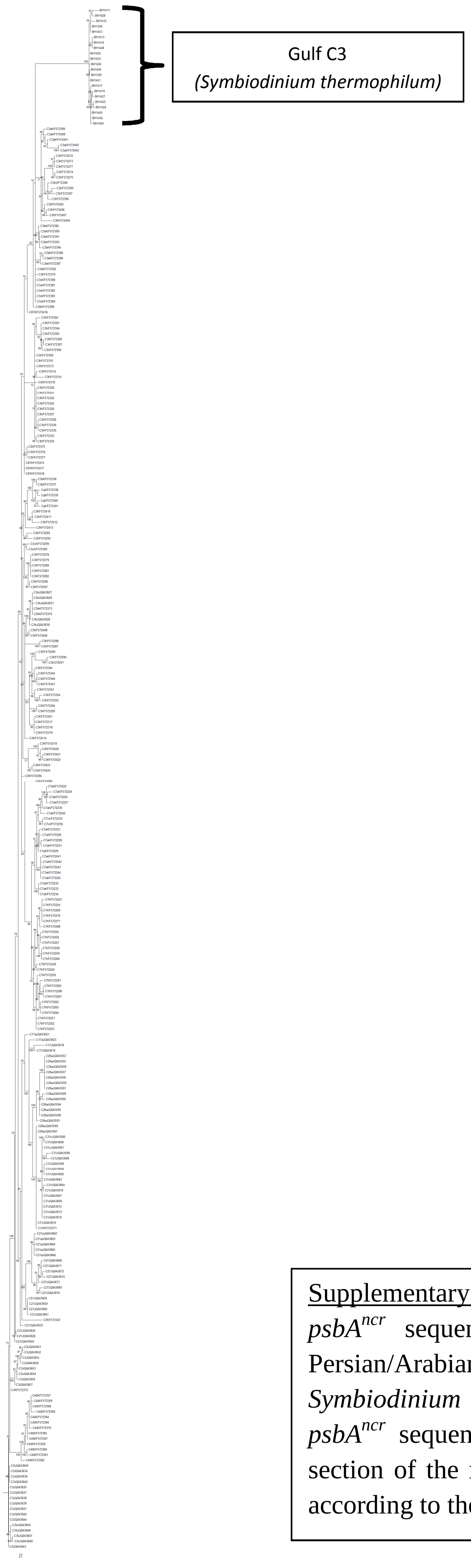

Supplementary Figure S3. Bayesian Inference phylogenies of *psbA*^{ncr} sequences from sites within and external to the Persian/Arabian Gulf. Estimated phylogeny of 22 *Symbiodinium thermophilum* and 264 reference (non-Gulf) *psbA*^{ncr} sequences. Tree creation is detailed in the Methods section of the main report. Non-Gulf sequences are annotated according to their ITS2 type and accession number.

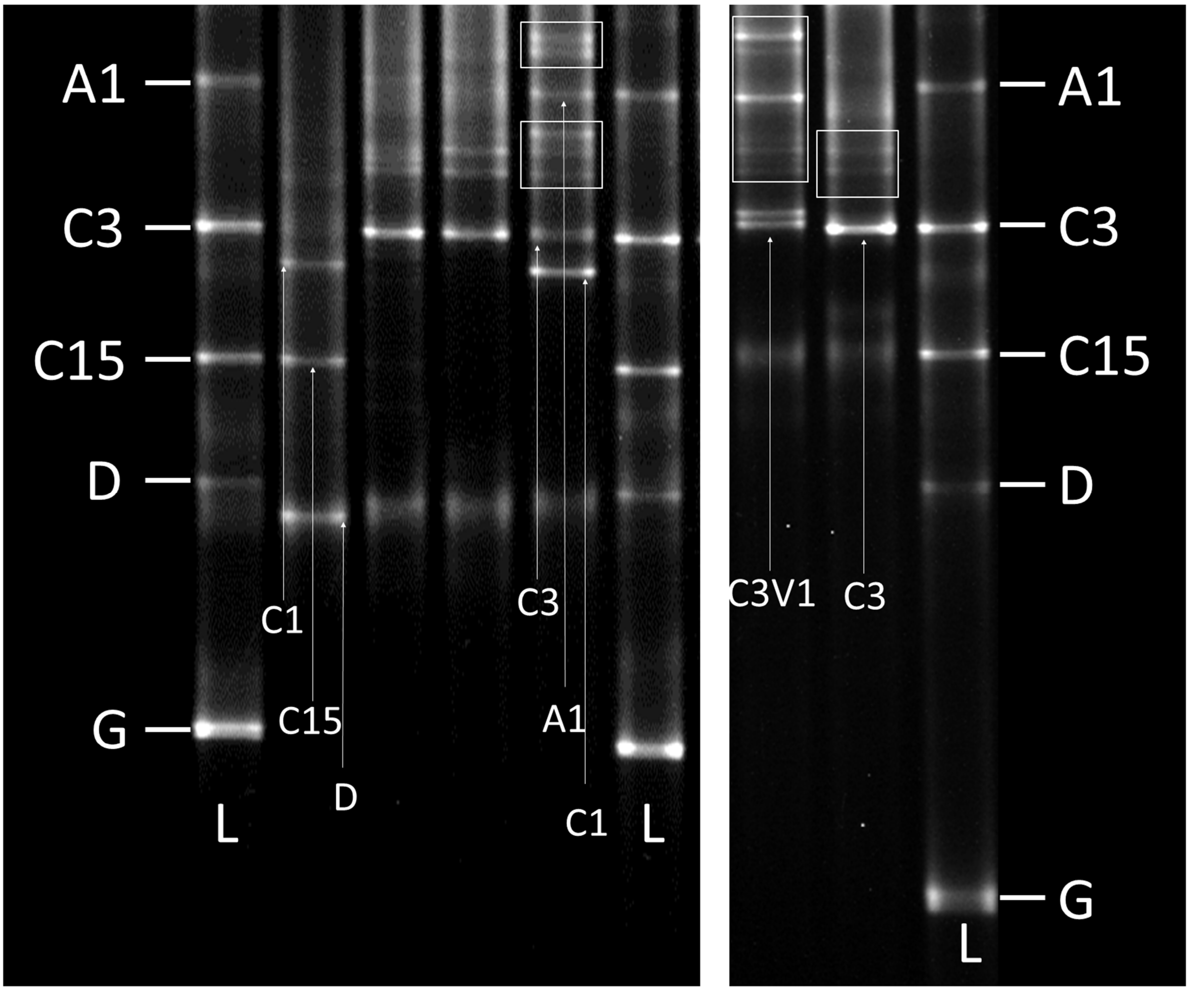

Supplementary Figure S4. Photographs of representative denaturing gradient gel electrophoresis (DGGE) gel demonstrating the resolution of predominant subclades found within southern Gulf corals in the 22 month seasonal analysis. Gel lanes with marker amplicons run in them are marked with L (Ladder) with bands annotated according to clade/subclade. The other lanes are examples of amplicons from DNA samples of individual coral colonies with predominant clades/subclades being annotated. Examples of bands representing alternative structural conformations, which, when excised, re-amplified and re-run on an identical gel form the same fingerprint as the original sample are highlighted by white rectangles.

Supplementary Tables

<u>Supplementary Table S1</u>. Dominant *Symbiodinium* ITS2 types attained by DGGE found in 6 Gulf coral species from Saadiyat reef. Each cell of the table represents one coral colony. Multiple symbiont types found in individual corals listed with their names separated by a forward slash. Multiple rows in cells of the table show the results for different tissue samples taken from the same colony.

	21.06.11	07.09.11	08.05.12	07.07.12	27.08.12	25.11.12	10.02.13	28.03.13
	'Summer'					'Winter'		
A. downingi	C3V1‡<br>C3V1	C3<br>C3V1<br>C3V1	Colony died					
	C3<br>C3/A1	C3<br>C3V1	C3V1	C3V1	C3V1	C3V1	C3V1/G	C3V1
	C3V1<br>C3V1<br>C3V1	C3V1<br>C3V1	C3V1<br>C3V1	C3V1<br>C3V1	C3V1<br>C3V1	C3V1	C3V1	C3V1
C. microphthalma	C3	C3	C3	C3	C3	C1<br>C3/D/C15<br>C3	C3/G<br>C3	C3/A1<br>C3/A1
	C3	C3	C3	C3	C3	C3<br>C3<br>C3/C1	C3/A1<br>C3/G	C3/A1<br>C3/A1<br>C3
	C3	C3	C3	C3/A1	C3/A1	C3/A1<br>C3/A1<br>C3/C15	C3/A1<br>C3<br>C3/G	C3<br>C3/A1<br>C3/A1
F. Pallida	C3/C15	C3/C15	C3/C15	C3/C15	C3/C15	C3/C15<br>C3/C15<br>C3/C15	C3/C15<br>C3	C3/C15<br>C3
	C3/A1/C15<br>C3/A1	C3/A1	C3/C15<br>C3/A1	C3/A1	C3/A1	C3/A1<br>C3/A1<br>C3/A1	C3/A1<br>C3/A1/C15<br>C3/G	C3/A1/C15
	C3/A1/C15	C3/C15/A1<br>C3/A1	C3/C15/A1	C3/C15/A1<br>C3	C3/A1/C15	C3/C15<br>C3	C3/C15<br>C3	C3/C15/A1
P. Daedalea	C3	C3	C3	C3	C3	C3/A1<br>C3<br>C3	C3<br>C3<br>C3	C3<br>C3<br>C3
	C3	C3	C3	C3	C3	C3/A1<br>C3<br>C3	C3/A1<br>C3/A1	C3<br>C3<br>C3
	C3	C3	C3	C3	C3	C3/A1<br>C3/A1<br>C3/A1	C3/A1<br>C3	C3<br>C3<br>C3
P. harrisoni	C3	C3	C3	C3	C3	C3	C3	C3
	C3	C3	C3	C3	C3	C3	C3	C3
	C3	C3	C3	C3	C3	C3	C3	C3
P. lutea	C3	C3	C3	C3	C3	C3	C3	C3
	C3	C3	C3	C3	C3	C3	C3	C3
	C3	C3	C3	C3	C3	C3	C3	C3

‡C3V1 refers to a novel ITS2 type variant (1bp different from ITS2 type C3) identified in this study.

Supplementary Table S2. Prevalence of the C3-*Gulf* ITS2 sequence variant found in Gulf C3 *Symbiodinium* populations in the southern Gulf. .

ID	Host species	Collection region	Collection date	No. of Seqs	C3-*Gulf*:C3
BH772	Porites harrisoni	Dalma	Sept. 2012	9	0:9
BH776	P. harrisoni	Dalma	Sept. 2012	9	0:9
BH1448	P. harrisoni	Dalma	Sept. 2012	9	3:6
BH1411	P. harrisoni	Dalma	Sept. 2012	9	3:6
BH787	P. harrisoni	Dalma	Sept. 2012	9	3:6
BH173	Porites lobata	Saadiyat	May 2010[†]	9	2:7
BH164	P. lobata	Saadiyat	May 2010[†]	13	3:10
BH167	P. lobata	Saadiyat	May 2010[†]	9	2:7
BH143	P. lobata	Saadiyat	May 2010[†]	17	3:14
BH213	P. lobata	Saadiyat	Oct. 2011	3	1:2
BH218	P. lobata	Saadiyat	Oct. 2011	7	1:6
BH223	P. lobata	Saadiyat	Oct. 2011	12	2:10
BH313	P. harrisoni	Saadiyat	Oct. 2011	3	1:2
BH318	P. harrisoni	Saadiyat	Oct. 2011	8	1:7
BH236	P. harrisoni	Saadiyat	Oct. 2011	7	2:5
BH377	P. harrisoni	Saadiyat	Sept. 2012	28	18:10
BH386	P. harrisoni	Saadiyat	Sept. 2012	28	16:12
BH403	P. harrisoni	Saadiyat	Sept. 2012	8	0:8
BH383	Porites lutea	Saadiyat	Sept. 2012	3	0:3
BH369	P. lobata	Saadiyat	Sept. 2012	15	2:13
BH1217	P. lobata	Saadiyat	Sept. 2012	6	1:5
BH1222	P. lobata	Saadiyat	Sept. 2012	2	1:1
BH1422	P. lutea	Saadiyat	Sept. 2012	9	7:2
BH1425	P. lutea	Umm Al Quwain	Mar. 2013	9	3:6
BH1426	P. lutea	Umm Al Quwain	Mar. 2013	18	3:15
BH1427	P. lutea	Umm Al Quwain	Mar. 2013	8	0:8
BH1392	P. lutea	Umm Al Quwain	Mar. 2013	8	0:8
BH293	Platygyra daedalea	Saadiyat	June 2011	3	1:2
BH302	P. daedalea	Saadiyat	June 2011	8	3:5
**Total**				**286**	**82:205**

[†] Zooxanthellae were sampled from corals from Saadiyat reef (collected May 2010) after four years of aquarium culture in the experimental mesocosm of Coral Reef Laboratory at the University of Southampton, UK[1]

Supplementary Table S3. GenBank accession numbers and corresponding ITS2 types of the reference sequences used in the *psbA*^{ncr} Bayesian Inference analysis. Sequences represent a subset of the sequences analysed in two previous analyses[2,3].

JQ043553	C26a	JQ043639	C3	KF572241	C7a	KF572294	C3	KF572359	C40	KF572411	C3
JQ043555	C26a	JQ043640	C3	KF572242	C7a	KF572295	C3	KF572360	C40	KF572412	C3
JQ043556	C26a	JQ043641	C3	KF572243	C7a	KF572296	C3	KF572361	C40	KF572413	C3
JQ043557	C26a	JQ043642	C3	KF572244	C7a	KF572297	C3	KF572362	C40	KF572414	C3
JQ043558	C26a	JQ043643	C3	KF572245	C7a	KF572299	C3c	KF572364	C40	KF572415	C87
JQ043559	C26a	JQ043644	C3	KF572246	C7a	KF572300	C3c	KF572365	C40	KF572416	C87
JQ043560	C26a	JQ043645	C3k	KF572247	C7	KF572301	C3	KF572366	C40	KF572417	C87
JQ043561	C26a	JQ043646	C3k	KF572248	C7	KF572302	C3	KF572367	C40	KF572418	C87
JQ043580	C31c	JQ043647	C3k	KF572249	C7	KF572303	C3	KF572368	C40	KF572419	C3
JQ043589	C26a	JQ043648	C3k	KF572250	C7	KF572304	C3	KF572369	C40	KF572420	C3
JQ043591	C26a	JQ043649	C3i	KF572251	C7	KF572305	C3	KF572370	C40	KF572421	C3
JQ043592	C26a	JQ043650	C3	KF572252	C7	KF572306	C3	KF572371	C31	KF572422	C3
JQ043593	C26a	JQ043651	C3i	KF572253	C7	KF572307	C3	KF572372	C3	KF572423	C3
JQ043594	C26a	JQ043652	C3	KF572254	C7	KF572308	C3	KF572373	C3h	KF572424	C3
JQ043595	C26a	JQ043653	C3	KF572255	C7	KF572309	C3	KF572374	C3h		
JQ043596	C26a	JQ043654	C3i	KF572256	C7	KF572310	C3	KF572375	C3		
JQ043598	C31	JQ043655	C3	KF572257	C7	KF572313	C3	KF572376	C3		
JQ043599	C31	JQ043656	C3	KF572258	C7	KF572314	C3	KF572377	C3		
JQ043600	C31	JQ043657	C3	KF572259	C7	KF572315	C3	KF572379	C3		
JQ043601	C31c	JQ043658	C21	KF572260	C7	KF572316	C3	KF572380	C3s		
JQ043602	C31	JQ043659	C21	KF572261	C7	KF572317	C3	KF572381	C3s		
JQ043603	C31	JQ043660	C21	KF572262	C7	KF572318	C3	KF572382	C3s		
JQ043604	C31	JQ043661	C21	KF572263	C7	KF572319	C3	KF572383	C3s		
JQ043606	C31	JQ043662	C21a	KF572264	C7	KF572320	C3	KF572384	C3s		
JQ043607	C31	JQ043663	C21a	KF572265	C7	KF572321	C3	KF572385	C3b		
JQ043608	C31	JQ043664	C21a	KF572266	C7	KF572322	C3	KF572386	C3b		
JQ043609	C31	JQ043665	C21a	KF572267	C7	KF572323	C3	KF572387	C3b		
JQ043610	C31	JQ043666	C21a	KF572268	C7	KF572324	C3	KF572388	C3b		
JQ043612	C31	JQ043668	C27	KF572269	C7	KF572325	C3	KF572389	C3b		
JQ043613	C31	JQ043669	C27	KF572270	C7	KF572326	C3	KF572390	C3b		
JQ043615	C31	JQ043670	C27	KF572271	C7	KF572327	C3	KF572391	C3b		
JQ043616	C31	JQ043671	C27	KF572272	C3	KF572328	C3	KF572392	C3b		
JQ043618	C17	JQ043672	C27	KF572273	C3	KF572329	C3	KF572393	C3b		
JQ043619	C17	JQ043673	C27	KF572274	C3	KF572330	C3	KF572394	C3b		
JQ043621	C17a	JQ043674	C27	KF572275	C3	KF572336	C3b	KF572395	C3		
JQ043623	C17a	KF572222	C7a	KF572277	C3	KF572337	C3b	KF572396	C3		
JQ043624	C21	KF572223	C7a	KF572278	C3	KF572338	Cq	KF572397	C3		
JQ043625	C21	KF572224	C7a	KF572279	C3	KF572339	Cq	KF572398	C3		
JQ043626	C21	KF572225	C7a	KF572280	C3	KF572340	Cq	KF572399	C3a		
JQ043627	C3h	KF572226	C7a	KF572281	C3	KF572341	Cq	KF572400	C3a		
JQ043628	C3h	KF572227	C7a	KF572282	C3	KF572343	C3	KF572401	C3a		
JQ043629	C3h	KF572228	C7a	KF572284	C3	KF572344	C3	KF572402	C3a		
JQ043630	C3h	KF572229	C7a	KF572285	C3	KF572345	C3	KF572403	C3a		
JQ043631	C3h	KF572230	C7a	KF572286	C3	KF572346	C3	KF572404	C3		
JQ043633	C21	KF572231	C7a	KF572287	C3	KF572347	C3	KF572405	C3		
JQ043634	C3	KF572232	C7a	KF572289	C3	KF572353	C3	KF572406	C3		
JQ043635	C3	KF572233	C7a	KF572290	C3	KF572354	C3	KF572407	C3		
JQ043636	C3	KF572234	C7a	KF572291	C3	KF572355	C3	KF572408	C3		
JQ043637	C3	KF572235	C7c	KF572292	C3	KF572357	C40	KF572409	C3		
JQ043638	C3	KF572236	C7c	KF572293	C3	KF572358	C40	KF572410	C3		

Supplementary Table S4. Details of the 22 Gulf *psbA*^{ncr} sequences used in the Bayesian Inference phylogenetic analyses. Underlined or asterisk-appended IDs represent sequences that underwent additional nrDNA ITS2 (see Supplementary Table S2) or both cp23S and *cob* molecular cloning, sequencing and analysis, respectively.

ID	Sampling Location	Host	ITS2 Type	Accession
BH1411*	Dalma	*Porites harrisoni*	C3	KM458273
BH1413	Dalma	*P. harrisoni*	C3	KM458274
BH1414*	Dalma	*P. harrisoni*	C3	KM458275
BH1415*	Dalma	*P. harrisoni*	C3	KM458276
BH1417	Saadiyat	*Porites lutea*	C3	KM458277
BH1419	Saadiyat	*Porites lobata*	C3	KM458278
BH1422*	Saadiyat	*P. lutea*	C3	KM458279
BH1423	Umm Al Quwain	*P. lutea*	C3	KM458280
BH1424	Umm Al Quwain	*P. lutea*	C3	KM458281
BH1425*	Umm Al Quwain	*P. lutea*	C3	KM458282
BH1426*	Umm Al Quwain	*P. lutea*	C3	KM458283
BH1427*	Umm Al Quwain	*P. lutea*	C3	KM458284
BH1428	Umm Al Quwain	*P. lutea*	C3	KM458285
BH1448*	Dalma	*P. harrisoni*	C3	KM458286
BH1449	Dalma	*P. lutea*	C3	KM458287
BH1450	Dalma	*P. lutea*	C3	KM458288
BH1451*	Saadiyat	*P. lobata*	C3	KM458289
BH1453*	Saadiyat	*P. lobata*	C3	KM458290
BH1456	Umm Al Quwain	*P. lutea*	C3	KM458291
BH1458	Umm Al Quwain	*P. lutea*	C3	KM458292
BH1356*	Saadiyat	*P. lutea*	C3	KM458293
BH1392*	Umm Al Quwain	*P. lutea*	C3	KM458294

Supplementary Table S5. Sequences utilised in the cp23S and *cob* phylogenies.

ITS2 type	Marker	Study	Accession number
C1	cp23S	Sampayo et al. 2009[4]	FJ529545
C3	cp23S	Sampayo et al. 2009	FJ529546
C35a	cp23S	Sampayo et al. 2009	FJ529548
C79	cp23S	Sampayo et al. 2009	FJ529549
C8a	cp23S	Sampayo et al. 2009	FJ529550
C78a	cp23S	Sampayo et al. 2009	FJ529551
C42a	cp23S	Sampayo et al. 2009	FJ529552
C33a	cp23S	Sampayo et al. 2009	FJ529553
C33	cp23S	Sampayo et al. 2009	FJ529554
C3nt	cp23S	Sampayo et al. 2009	FJ529555
C91	cp23S	Pochon et al. 2012[5]	JN557980
C90	cp23S	Pochon et al. 2012	JN557975
C15	cp23S	Pochon et al. 2012	JN557974
Gulf C3	cp23S	This study	KP234523
C1	*cob*	Sampayo et al. 2009	FJ529534
C3	*cob*	Sampayo et al. 2009	FJ529535
C35a	*cob*	Sampayo et al. 2009	FJ529537
C79	*cob*	Sampayo et al. 2009	FJ529538
C8a	*cob*	Sampayo et al. 2009	FJ529539
C78a	*cob*	Sampayo et al. 2009	FJ529540
C42a	*cob*	Sampayo et al. 2009	FJ529541
C33a	*cob*	Sampayo et al. 2009	FJ529542
C33	*cob*	Sampayo et al. 2009	FJ529543
C3nt	*cob*	Sampayo et al. 2009	FJ529544
C91	*cob*	Pochon et al. 2012	JN557946
C90	*cob*	Pochon et al. 2012	JN557945
C15	*cob*	Pochon et al. 2012	JN557944
Gulf C3	*cob*	This study	KP234522

Supplementary Table S6. A list of Primers and thermal cycles used in PCR to amplify amplicons used as part of this study. Thermal cycles are only listed where they differ from those used in the referenced study associated with each primer pair. Reaction conditions for all PCRs were as used for primer pair SYM_VAR_FWD & SYM_VAR_REV in Hume et al. 2013[6].

Amplicon	Primers	Thermal Cycle
ITS1-5.8S-ITS2		
	SYM_VAR_FWD[6]	
	SYM_VAR_REV[6]	
ITS2-DGGE		95°C 2min
	SYM_VAR_5.8S2<br>5'GAATTGCAGAACTCCGTGAACC3'	30x[95°C 30s, 56°C 30s, 72°C 30s]
	SYM_VAR_CLAMP<br>5'[CGCCCGCCGCGCCCCGCGCCCGTCCC-GCCGCCCCCGCCC] CGGGTTCTCTTGTT-TGACTTCATGC3'	72°C 5min
psbA^{ncr}		
	psbAFor_1[3]	
	psbARev_1[3]	
cp23S domain V		
	23S1M13[4]	
	23S2M13[4]	
Cytochrome b		95°C 2min
	cob1 (forward)[7]	40x[95°C 20s, 55°C 45s, 72°C 2min]
	dinocob1 (reverse)[7]	72°C 10min

Supplementary Methods

Details of the DGGE protocol

Denaturing gradient gel electrophoresis (DGGE) analysis was used to identify the predominant zooxanthellae ITS2 types present in each coral colony analysed in this study. All visible bands on DGGE gels were characterised according to their predominant ITS2 sequence as described below. Bands characterised by novel ITS2 sequences (not previously characterised as a representative of an ITS2 type according to previous DGGE analyses) were considered to be intragenomic variants unless: the band ran as the brightest band, or if no band representing a closely related sequence (1-2 bp difference) was apparent in the same sample. If rare sequence variants were identified during characterisation of a single band these sequences were deemed to be representative of intragenomic variants. Intragenomic variants were excluded from the study. For each sample to be run in the DGGE analysis the complete ITS1-5.8S-ITS2 region of the nrDNA was first amplified using primers SYM_VAR_FWD and SYM_VAR_REV as described previously[6]. The PCR was then run on a 0.8% agarose gel, the product size verified, the PCR product cut out (using a sterilised scalpel) and homogenised using a sterile micropestle in 500µl of deionised water. This sample was then stored at -20°C. 3µl of this homogenised sample was then used as a template for a DGGE specific PCR using primers SYM_VAR_5.8SII 5’GAAT-TGCAGAACTCCGTGAACC3’ and SYM_VAR_Clamp 5’[CGCCCGCCGCGCCCCGCG-CCCGTCCCGCCGCCCCCGCCC] CGGGTTCTCTTGTTTGACTTCATGC3’ (identical to SYM_VAR_REV but with the addition of a 40bp GC clamp). These primers amplify a partial region of the 5.8S, the complete ITS2 region and a partial region of the 28S and were used with reaction conditions identical to those with the SYM_VAR_FWD and SYM_VAR_REV primer pair with thermal cycling of 95°C for 2mins, 30 cycles of 95°C for 30s, 56°C for 30s and 72°C for 30s followed by a final extension time of 72°C for 5 minutes.

DGGE analyses were conducted using a BioRad DCode System for DGGE with a model 475 gradient former. 10 µl of PCR product was loaded onto an 8% gel with a 32.5-57.5% gradient (the 100% denaturing solution was made according to the manufacturers instruction) using an equal mix of 2 x loading dye (made according to the manufacturer's instructions). The gel was run for 1400Vh. Gels were stained (with a final concentration of 50µg/ml of ethidium bromide) and destained for 15 min in 200ml of TAE running buffer. Markers for ITS2 sequences were created by amplifying cloned plasmid DNA templates that contained previously sequenced and characterised ITS2 nrDNA sequences. When amplifying DGGE fragments from plasmid template, an annealing temperature of 66°C was used with 28 cycles. The presence of *Symbiodinium* types within a coral were characterised by analysing the unique 'fingerprint' produced. This fingerprint was characteristically made up of a single bright band and a multitude of fainter bands. When excised, reamplified and re-run, many of the fainter bands produced the same fingerprint (including the bright and fainter bands) as the original sample. In this case, these bands were considered to be structural conformations of the ITS2 sequence and part of the unique fingerprint of the sample. Over cycling of PCR products was avoided to prevent the formation of excessive structural conformations. Bands that were excised, re-amplified, re-run and produced only a single bright band, that ran to the point where the band had originally been excised were taken as indicators of genuine ITS2 sequences (rather than structural conformations). In cases where samples were run and produced fingerprints that differed from any previously run samples, all of the bands were excised, re-amplified and re-run in order to characterise them. Any bands characteristic of a genuine ITS2 sequence, rather than a structural conformation, were excised from the gel and homogenised in 500µl of deionised water. 3µl of this homogenate was then used as template to re-amplify the band's ITS2 sequence using high fidelity Advantage 2 PCR mix polymerase (Clontech) and primers SYM_VAR_5.8SII and SYM_VAR_REV with reaction conditions

and thermal cycling identical to that of the SYM_VAR_5.8SII and SYM_VAR_Clamp primer pair. This PCR product was then molecularly cloned and sequenced as detailed in the methods section for the other ITS2 sequences analysed in this study. The DGGE procedure utilised in this study had been used to successfully characterise subclades, A1, C3, C1, multiple C3 variants, C15, multiple C15 variants, D1-4 and clade G. To ensure a lack of bias in the PCR amplification between clades, equal amounts of clade A and clade D template were amplified using the DGGE primers and run on a gel. Two major bands of equal intensity were produced representative of each clade, thus indicating a suitable lack of PCR bias. Despite the pseudo-quantitative nature of the DGGE analysis, subclades were only characterised as present or absent in this study and no attempt at quantification was made according to band intensity.

Supplementary References

1. D'Angelo, C. & Wiedenmann, J. An experimental mesocosm for long-term studies of reef corals. *J. Mar. Biol. Assoc. UK* **92,** 769–775 (2012).

2. Thornhill, D. J., Lewis, A. M., Wham, D. C. & Lajeunesse, T. C. Host-specialist lineages dominate the adaptive radiation of reef coral endosymbionts. *Evolution* **68,** 352–367 (2014).

3. LaJeunesse, T. C. & Thornhill, D. J. Improved resolution of reef-coral endosymbiont (*Symbiodinium*) species diversity, ecology, and evolution through psbA non-coding region genotyping. *PLoS ONE* **6,** e29013 (2011).

4. Sampayo, E. M., Dove, S. & LaJeunesse, T. C. Cohesive molecular genetic data delineate species diversity in the dinoflagellate genus *Symbiodinium*. *Mol. Ecol.* **18,** 500–519 (2009).

5. Pochon, X., Putnam, H. M., Burki, F. & Gates, R. D. Identifying and characterizing alternative molecular markers for the symbiotic and free-living dinoflagellate genus *Symbiodinium*. *PLoS ONE* **7,** e29816 (2012).

6. Hume, B. *et al.* Corals from the Persian/Arabian Gulf as models for thermotolerant reef-builders: prevalence of clade C3 *Symbiodinium,* host fluorescence and *ex situ* temperature tolerance. *Mar. Pollut. Bull.* **72,** 313–22 (2013).

7. Zhang, H., Bhattacharya, D. & Lin, S. Phylogney of dinoflagellates based on mitochondrial cytochrome b and nuclear small subunit rDNA sequence comparisons. *J. Phycol.* **41,** 411–420 (2005).
